# Bis[4-(2-carbamoylhydrazin-1-yl­idene-κ^2^ *N* ^1^,*O*)-5-hydroxy­methyl-2-methyl­pyridinium-3-olato-κ*O* ^3^]cobalt(II) dinitrate dihydrate

**DOI:** 10.1107/S1600536810003570

**Published:** 2010-03-13

**Authors:** Dragoslav Vidovic, Violeta Jevtovic

**Affiliations:** a Chemistry Research Laboratory, Chemistry Department, University of Oxford, Mansfield Road, Oxford OX1 3TA, England; b Department of Chemistry, Faculty of Sciences, University of Novi Sad, 21000 Novi Sad, Trg D. Obradovica 3, Serbia

## Abstract

The asymmetric unit of the title compound, [Co(C_9_H_12_N_4_O_3_)_2_](NO_3_)_2_·2H_2_O, consists of a discrete cationic [Co(PLSC)_2_]^2+^ complex unit [PLSC is 4-(2-carbamoylhydrazin-1-yl­idene)-5-hydroxy­methyl-2-methyl­pyridinium-3-ol­ato], two NO_3_^−^ and two water mol­ecules. The two tridentate PLSC ligands of the cation are zwitterions related to each other by a non-crystallographic *C*_2_ axis. The Co^II^ ion is in a disorted octa­hedral coordination environment. The crystal structure is composed of alternating NO_3_/H_2_O and complex layers supported by extensive C—H⋯O, N—H⋯O and N—H⋯N hydrogen bonding.

## Related literature

For the preparation and structure of other complexes incorporating PLSC ligands, see for example: Poleti *et al.* (2003 ▶); Leovac *et al.* (2007*a* ▶); Jacimovic *et al.* (2007 ▶); Knezevic *et al.* (2003 ▶). For the preparation and structures of similar complexes incorporating thio­semicarbazone (TSC) ligands, see: Belicchi Ferrari *et al.* (1998 ▶); Leovac *et al.* (2007*b* ▶). For background to the biological acitiviy of semicarbazones and thio­semicarbazones, see: West *et al.* (1991 ▶). For puckering parameters, see: Cremer & Pople (1975 ▶). For the Chebychev weighting scheme, see: Prince (1982 ▶); Watkin (1994 ▶).
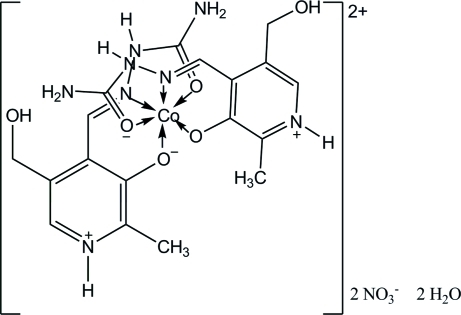


## Experimental

### 

#### Crystal data

[Co(C_9_H_12_N_4_O_3_)_2_](NO_3_)_2_·2H_2_O
*M* *_r_* = 667.41
Monoclinic, 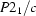

*a* = 11.0358 (1) Å
*b* = 18.4859 (2) Å
*c* = 13.8380 (1) Å
β = 106.5705 (6)°
*V* = 2705.80 (4) Å^3^
*Z* = 4
Mo *K*α radiation
μ = 0.72 mm^−1^
*T* = 150 K
0.38 × 0.08 × 0.03 mm

#### Data collection

Nonius KappaCCD diffractometer
Absorption correction: multi-scan *DENZO*/*SCALEPACK* (Otwinowski & Minor, 1997 ▶) *T*_min_ = 0.92, *T*_max_ = 0.98
38405 measured reflections
5212 independent reflections
4121 reflections with *I* > 2σ(*I*)
*R*_int_ = 0.056

#### Refinement

*R*[*F* ^2^ > 2σ(*F* ^2^)] = 0.033
*wR*(*F* ^2^) = 0.041
*S* = 1.14
5212 reflections
389 parameters
H-atom parameters constrained
Δρ_max_ = 0.56 e Å^−3^
Δρ_min_ = −0.42 e Å^−3^

### 

Data collection: *COLLECT* (Nonius, 2001 ▶); cell refinement: *DENZO*/*SCALEPACK* (Otwinowski & Minor, 1997 ▶); data reduction: *DENZO*/*SCALEPACK*; program(s) used to solve structure: *SIR92* (Altomare *et al.*, 1994 ▶); program(s) used to refine structure: *CRYSTALS* (Betteridge *et al.*, 2003 ▶); molecular graphics: *CAMERON* (Watkin *et al.*, 1996 ▶) and *SHELXTL* (Sheldrick, 2008 ▶); software used to prepare material for publication: *CRYSTALS*.

## Supplementary Material

Crystal structure: contains datablocks I, global. DOI: 10.1107/S1600536810003570/lh2980sup1.cif

Structure factors: contains datablocks I. DOI: 10.1107/S1600536810003570/lh2980Isup2.hkl

Additional supplementary materials:  crystallographic information; 3D view; checkCIF report

## Figures and Tables

**Table 1:** Hydrogen-bond geometry (Å, °)

*D*—H⋯*A*	*D*—H	H⋯*A*	*D*⋯*A*	*D*—H⋯*A*
C7—H71⋯O11	0.94	2.33	2.992 (3)	127
C17—H172⋯O11^i^	0.97	2.58	3.331 (3)	135
C22—H221⋯O2^ii^	0.93	2.35	3.153 (3)	144
N27—H271⋯O35^iii^	0.86	2.18	2.950 (3)	149
N27—H271⋯N36^iii^	0.86	2.57	3.400 (3)	163
N27—H271⋯O37^iii^	0.86	2.27	2.995 (3)	142
C30—H301⋯O37^iv^	0.97	2.49	3.369 (3)	151
O11—H325⋯O16^i^	0.82	2.00	2.783 (3)	159
O11—H325⋯O18^i^	0.82	2.59	3.132 (3)	125
O39—H11⋯O40	0.84	2.01	2.851 (3)	175
O31—H17⋯O42^v^	0.81	1.95	2.718 (3)	158
C17—H23⋯O40	0.96	2.59	3.391 (3)	141
O39—H45⋯O31^vi^	0.85	2.05	2.892 (3)	171
O34—H7⋯O11^vii^	0.83	1.96	2.767 (3)	164
O34—H19⋯O25	0.83	2.06	2.852 (3)	158
N13—H131⋯O40	0.75	2.22	2.963 (3)	169
N13—H131⋯O42	0.75	2.38	2.971 (3)	136
N5—H2⋯O38^i^	0.86	1.93	2.789 (3)	174
N4—H3⋯O37^viii^	0.86	2.09	2.948 (3)	174
N33—H4⋯O39^ix^	0.86	2.08	2.893 (3)	158
N33—H5⋯O34^ii^	0.88	2.06	2.874 (3)	153
N4—H6⋯O35^i^	0.88	2.09	2.967 (3)	174
N20—H9⋯O34^ii^	0.86	2.21	2.946 (3)	143

Symmetry codes: (i) 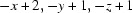; (ii) 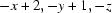; (iii) 
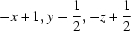
; (iv) 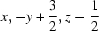; (v) 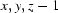; (vi) 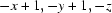; (vii) 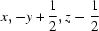; (viii) 
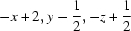
; (ix) 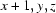.

**Fig. 1.**
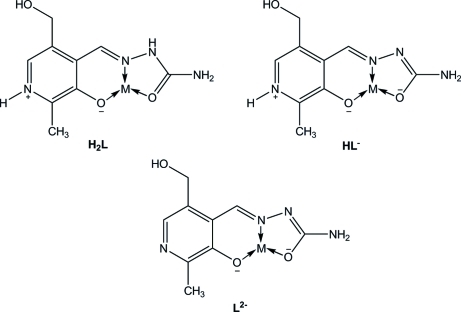
Different forms of the PLSC ligand.

**Fig. 2.**
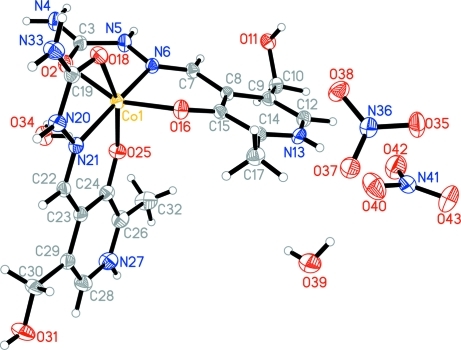
The asymmetric unit of 1. The thermal ellipsoids are drawn at the 50% probability level. H atoms are not shown.

**Fig. 3.**
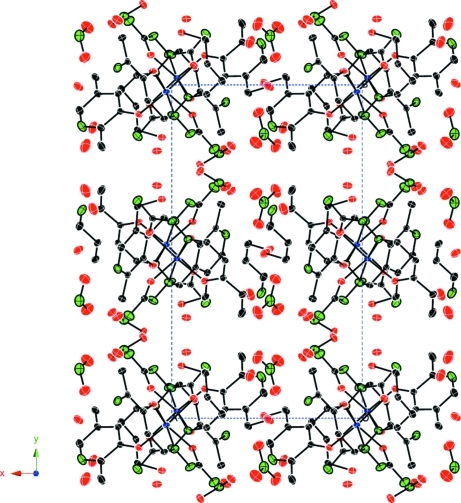
Crystal packing diagram of 1 viewed along the z axis showing the layered motif. H atoms are not shown.

## supplementary crystallographic information

### Comment

Semicarbazones (SC) and thiosemicarbazones (TSC) are excellent chelating ligands of different denticity that possess a broad range of biological activity as antifungal, anti-viral, anti-malarian and anti-tumour agents (West *et al.*, 1991). The synthetic, structural as well as the biological activity of TSC-based ligands have been explored to a greater extent than their SC-based analogues. In fact, only a handful of reports have been published revealing the syntheses and structures of complexes incorporating 3–hydroxy–5–hydroxymethyl–2–methyl– pyridine–4–carbaldehyde semicarbazone (PLSC) ligand (see for example Knezevic *et al.*, 2003) and all the reports describe complexes incorporating one PLSC ligand except for a report by Leovac *et al.*, (2007b), which includes the synthetic and structural descriptions of a complex with two PTSC ligands in its coordination sphere. It is worth noting that PLSC ligand can adopt three different forms in the coordination sphere of a transition metal namely neutral (but zwitterionic) H_2_L, monoanionic HL^-^ (pyridinium deprotonation) and dianionic L^2-^ (both pyridinium and hydrazine deprotonation) forms (see Fig. 1).

Herein, we report the second PLSC-based complex ([Co(H_2_L)_2_].2NO_3_.2H_2_O, 1 (Figure 2), which contains two PLSC ligands in its coordination sphere. However, complex 1 includes both PLSC ligands in their neutral zwitterionic forms while in the corresponding complex reported by Leovac *et al.*, (2007b) both ligands are in their monodeprotonated forms HL^-^. Thus, the title complex 1 is the first bis-PLSC-based complex that contains neutral PLSC ligands.

The molecular structure for 1 is shown in Fig. 2 and it contains a discrete dicationic unit [Co(H_2_L)_2_]^2+^, two NO_3_^-^ anions and two H_2_O molecules. The crystal structure is connected by an extensive hydrogen-bonding network. The structure is best described as layered (Fig. 3) in which one layer consists of [Co(H_2_L)_2_]^2+^ units connected by alternating O16—H···O11 and C22—H···O2 hydrogen bonds additionally stabilized by a water molecule. The other layer consists of NO_3_^-^ anions and water molecules that also form several hydrogen bonds with the first layer.

The values for certain bond lengths and angles of the ligand backbone (O(phenolic)–C–C–C–N (hydrazine)–*N*–*C*–*O*(carbonyl)) are crystallographic evidence used to determine which form (H_2_L, HL^-^ or L_2_^-^) the PLSC ligand adopts once coordinated to a metal centre. For example, the first deprotonation of H_2_L, forming monoanionic form HL^-^, changes the carbonyl C—O from a double to a single bond and the hydrazine N—N from a single to a double bond. Further deprotonation to form L_2_^-^ leads to a change in the C—N—C angle of the pyridine ring from 125° to 118°. Thus, the carbonyl C–O (1.249 (3) and 1.295 (3) Å) and N–N (1.368 (2) and 1.375 (2) Å) bond lengths, and the pyridine C–N–C angles (124.9 (2) Å and 124.19 (19) °) are the concrete evidence that the PTSC ligands are in their neutral (H_2_L) forms in complex 1.

The environment around the central cobalt cation in 1 can be best described as a distorted octahedral geometry. In fact, the N6–Co–N21 angle (171.12 (7)°) is somewhat similar to the theoretical 180° , but the other two, symmetry-related, O2–Co1–O16 (154,44 (7)° ), and O18–Co1–O25 (162.82 (6)° ) angles greatly deviate from linearity due to the chelation rings strain. Furthermore, the angles formed by the phenolic and carbonyl O atoms and Co (O2–Co1–O18 and O16–Co1–O25) differ from 90° (83.21 (6) and 101.92 (7)°, respectively) confirming a distorted octahedral geometry around the central Co cation.

The ring-puckering parameters defined for the atom sequence O16(O25)–C15(C24)–C8(C23)– C7(C22)–N6(N21)–Co are Θ = 64.8 (7)°, Q1=0.142 (2) Å and Q2= 0.067 (2) Å corresponding to a twist (^1^T_2_) conformation while the six-membered ring has and a total puckering amplitude of 0.1572 (18) Å corresponding to a skew (1S2) conformation (Cremer & Pople , 1975). The O16(25) atoms are the only atoms in the chelate ligand which exhibit strong H bonding interactions with O11 of the NO_3_^-^ anions. The geometry of NO_3_^-^ groups do not deviate from the usual literature values and the summary of the H bonding (O–H···O, N—H···O and C—H···O) in 1 are given in Table 1.

### Experimental

The title complex was prepared by the reaction of Co(NO_3_)_2_ .6H_2_O and PLSC in a 1:1 molar ratio, using warm H_2_O as the solvent. Brown single crystals of 1 were obtained after allowing the reaction mixture to stand overnight.

### Refinement

The H atoms were all located in a difference map, but those attached to carbon atoms were repositioned geometrically. The H atoms were initially refined with soft restraints on the bond lengths and angles to regularise their geometry (C—H in the range 0.93–0.98, N—H in the range 0.86–0.89 N—H to 0.86 O—H = 0.82 Å) and U_iso_(H) (in the range 1.2-1.5 times U_eq_ of the parent atom), after which the positions were refined with riding constraints.

A three term Chebychev polynomial weighting scheme was applied (Watkin, 1994; Prince, 1982).

### Crystal data

**Table:**

[Co(C_9_H_12_N_4_O_3_)_2_](NO_3_)_2_·2H_2_O	*F*(000) = 1380
*M**_r_* = 667.41	*D*_x_ = 1.638 Mg m^−^^3^
Monoclinic, *P*2_1_/*c*	Mo *K*α radiation, λ = 0.71073 Å
Hall symbol: -P 2ybc	Cell parameters from 6300 reflections
*a* = 11.0358 (1) Å	θ = 5–27°
*b* = 18.4859 (2) Å	µ = 0.72 mm^−^^1^
*c* = 13.8380 (1) Å	*T* = 150 K
β = 106.5705 (6)°	Block, green
*V* = 2705.80 (4) Å^3^	0.38 × 0.08 × 0.03 mm
*Z* = 4	

### Data collection

**Table:**

Nonius KappaCCD diffractometer	4121 reflections with *I* > 2σ(*I*)
graphite	*R*_int_ = 0.056
ω scans	θ_max_ = 27.5°, θ_min_ = 5.1°
Absorption correction: multi-scan *DENZO*/*SCALEPACK* (Otwinowski & Minor, 1997)	*h* = −14→14
*T*_min_ = 0.92, *T*_max_ = 0.98	*k* = −23→23
38405 measured reflections	*l* = −17→17
6133 independent reflections	

### Refinement

**Table:**

Refinement on *F*	Hydrogen site location: inferred from neighbouring sites
Least-squares matrix: full	H-atom parameters constrained
*R*[*F*^2^ > 2σ(*F*^2^)] = 0.033	Method, part 1, Chebychev polynomial, (Watkin, 1994, *P*rince, 1982) [*w*eight] = 1.0/[A_0_*T_0_(x) + A_1_*T_1_(x) ··· + A_n-1_]*T_n-1_(x)] where A_i_ are the Chebychev coefficients listed belo*w* and x = *F* /*F*max Method = Robust Weighting (*P*rince, 1982) W = [*w*eight] * [1-(delta*F*/6*sigma*F*)^2^]^2^ A_i_ are: 0.444 0.165 0.244
*wR*(*F*^2^) = 0.041	(Δ/σ)_max_ = 0.001
*S* = 1.14	Δρ_max_ = 0.56 e Å^−^^3^
5212 reflections	Δρ_min_ = −0.42 e Å^−^^3^
389 parameters	Extinction correction: Larson (1970), Equation 22
0 restraints	Extinction coefficient: 80 (17)
Primary atom site location: structure-invariant direct methods	

### Fractional atomic coordinates and isotropic or equivalent isotropic displacement parameters (Å^2^)

**Table:**

	*x*	*y*	*z*	*U*_iso_*/*U*_eq_	
Co1	0.97214 (3)	0.479038 (15)	0.21508 (2)	0.0178	
O2	1.10949 (15)	0.41005 (8)	0.18293 (11)	0.0221	
C3	1.1436 (2)	0.35913 (12)	0.24402 (16)	0.0197	
N4	1.22358 (19)	0.30776 (11)	0.23500 (15)	0.0266	
N5	1.09857 (18)	0.35397 (10)	0.32629 (14)	0.0237	
N6	1.01658 (17)	0.40702 (10)	0.33633 (13)	0.0189	
C7	0.9609 (2)	0.39890 (12)	0.40564 (16)	0.0191	
C8	0.8744 (2)	0.45254 (11)	0.42558 (16)	0.0183	
C9	0.8127 (2)	0.43675 (12)	0.50011 (16)	0.0200	
C10	0.8221 (2)	0.36423 (12)	0.55277 (17)	0.0232	
O11	0.94816 (15)	0.34273 (8)	0.60528 (11)	0.0235	
C12	0.7357 (2)	0.48771 (12)	0.52394 (17)	0.0229	
N13	0.71667 (17)	0.55170 (10)	0.47433 (14)	0.0226	
C14	0.7688 (2)	0.56970 (12)	0.40180 (16)	0.0201	
C15	0.85115 (19)	0.52005 (12)	0.37386 (15)	0.0186	
O16	0.90135 (15)	0.54119 (8)	0.30417 (12)	0.0230	
C17	0.7403 (2)	0.64164 (12)	0.35176 (18)	0.0256	
O18	1.11612 (15)	0.55823 (9)	0.23239 (11)	0.0246	
C19	1.1000 (2)	0.59940 (12)	0.15863 (16)	0.0195	
N20	0.99748 (17)	0.59111 (10)	0.07567 (14)	0.0211	
N21	0.91416 (17)	0.53710 (9)	0.08134 (13)	0.0187	
C22	0.8162 (2)	0.52824 (12)	0.00484 (16)	0.0191	
C23	0.7192 (2)	0.47389 (12)	0.00103 (16)	0.0197	
C24	0.7303 (2)	0.42092 (12)	0.07771 (17)	0.0212	
O25	0.82772 (15)	0.41181 (8)	0.15522 (12)	0.0231	
C26	0.6286 (2)	0.37144 (13)	0.06785 (18)	0.0271	
N27	0.52905 (19)	0.37585 (12)	−0.01369 (16)	0.0308	
C28	0.5171 (2)	0.42374 (14)	−0.08929 (18)	0.0288	
C29	0.6111 (2)	0.47329 (13)	−0.08471 (16)	0.0225	
C30	0.5993 (2)	0.52333 (13)	−0.17260 (16)	0.0253	
O31	0.49006 (17)	0.50463 (10)	−0.25181 (14)	0.0361	
C32	0.6314 (3)	0.31578 (15)	0.1459 (2)	0.0380	
N33	1.17911 (19)	0.65324 (11)	0.15436 (15)	0.0266	
O34	0.92284 (17)	0.30192 (9)	0.05176 (13)	0.0298	
O35	0.71622 (18)	0.80490 (10)	0.60566 (13)	0.0335	
N36	0.75110 (18)	0.79336 (10)	0.52883 (14)	0.0225	
O37	0.68648 (16)	0.81769 (9)	0.44575 (12)	0.0291	
O38	0.85047 (15)	0.75880 (9)	0.53404 (13)	0.0298	
O39	0.41254 (18)	0.62823 (12)	0.31113 (15)	0.0467	
O40	0.5576 (2)	0.67311 (12)	0.50711 (15)	0.0484	
N41	0.51580 (19)	0.64733 (13)	0.57582 (15)	0.0311	
O42	0.5441 (2)	0.58280 (11)	0.59929 (16)	0.0453	
O43	0.4521 (2)	0.68264 (14)	0.61762 (18)	0.0566	
H71	0.9774	0.3573	0.4460	0.0228*	
H101	0.7720	0.3664	0.6016	0.0279*	
H102	0.7876	0.3265	0.5016	0.0267*	
H121	0.6942	0.4791	0.5765	0.0442*	
H172	0.8136	0.6724	0.3747	0.0384*	
H173	0.7197	0.6360	0.2794	0.0387*	
H221	0.8066	0.5589	−0.0502	0.0232*	
H271	0.4677	0.3462	−0.0184	0.0362*	
H301	0.5918	0.5725	−0.1507	0.0287*	
H302	0.6725	0.5194	−0.1970	0.0290*	
H321	0.5552	0.2872	0.1270	0.0565*	
H322	0.6385	0.3400	0.2085	0.0563*	
H323	0.7039	0.2852	0.1537	0.0561*	
H325	0.9802	0.3770	0.6409	0.0357*	
H11	0.4517	0.6415	0.3701	0.0682*	
H17	0.4888	0.5336	−0.2964	0.0494*	
H23	0.6697	0.6643	0.3664	0.0389*	
H45	0.4494	0.5912	0.2972	0.0683*	
H7	0.9433	0.2609	0.0759	0.0456*	
H19	0.9005	0.3256	0.0950	0.0455*	
H324	0.4444	0.4228	−0.1444	0.0326*	
H131	0.6742	0.5791	0.4884	0.0126*	
H2	1.1172	0.3178	0.3671	0.0294*	
H3	1.2527	0.3079	0.1833	0.0331*	
H4	1.2454	0.6582	0.2043	0.0307*	
H5	1.1634	0.6795	0.0993	0.0304*	
H6	1.2462	0.2740	0.2810	0.0331*	
H9	0.9850	0.6186	0.0235	0.0258*	

### Atomic displacement parameters (Å^2^)

**Table:**

	*U*^11^	*U*^22^	*U*^33^	*U*^12^	*U*^13^	*U*^23^
Co1	0.02145 (15)	0.01664 (15)	0.01580 (14)	0.00014 (12)	0.00608 (10)	0.00114 (12)
O2	0.0268 (8)	0.0207 (8)	0.0212 (8)	0.0020 (6)	0.0105 (6)	0.0033 (6)
C3	0.0217 (11)	0.0196 (10)	0.0184 (10)	−0.0021 (8)	0.0069 (8)	−0.0006 (8)
N4	0.0329 (11)	0.0264 (10)	0.0243 (10)	0.0087 (9)	0.0145 (8)	0.0036 (8)
N5	0.0311 (10)	0.0208 (9)	0.0223 (9)	0.0101 (8)	0.0127 (8)	0.0064 (8)
N6	0.0211 (9)	0.0188 (9)	0.0178 (9)	0.0039 (7)	0.0071 (7)	0.0009 (7)
C7	0.0238 (11)	0.0176 (10)	0.0164 (10)	0.0019 (8)	0.0062 (8)	0.0022 (8)
C8	0.0186 (10)	0.0196 (10)	0.0167 (10)	−0.0011 (8)	0.0050 (8)	−0.0021 (8)
C9	0.0193 (10)	0.0224 (11)	0.0185 (10)	−0.0027 (9)	0.0057 (8)	−0.0014 (9)
C10	0.0253 (11)	0.0230 (11)	0.0237 (11)	−0.0030 (9)	0.0107 (9)	0.0013 (9)
O11	0.0276 (8)	0.0198 (7)	0.0226 (8)	−0.0004 (6)	0.0064 (6)	−0.0011 (6)
C12	0.0226 (11)	0.0268 (11)	0.0211 (11)	−0.0017 (9)	0.0091 (9)	0.0013 (9)
N13	0.0198 (9)	0.0240 (9)	0.0254 (10)	0.0046 (8)	0.0089 (8)	−0.0033 (8)
C14	0.0191 (10)	0.0209 (10)	0.0191 (10)	−0.0022 (8)	0.0036 (8)	−0.0036 (8)
C15	0.0195 (10)	0.0194 (10)	0.0167 (10)	−0.0013 (9)	0.0052 (8)	−0.0021 (9)
O16	0.0298 (8)	0.0180 (7)	0.0241 (8)	0.0012 (6)	0.0126 (7)	0.0028 (6)
C17	0.0278 (12)	0.0213 (11)	0.0279 (12)	0.0040 (9)	0.0083 (9)	0.0009 (9)
O18	0.0267 (8)	0.0256 (8)	0.0206 (8)	−0.0039 (7)	0.0052 (6)	0.0002 (7)
C19	0.0205 (10)	0.0186 (10)	0.0202 (10)	−0.0003 (8)	0.0069 (8)	−0.0015 (8)
N20	0.0225 (9)	0.0188 (9)	0.0213 (9)	−0.0057 (7)	0.0052 (7)	0.0038 (7)
N21	0.0206 (9)	0.0167 (9)	0.0203 (9)	−0.0014 (7)	0.0080 (7)	−0.0002 (7)
C22	0.0209 (10)	0.0203 (10)	0.0173 (10)	0.0004 (9)	0.0073 (8)	0.0014 (8)
C23	0.0200 (10)	0.0202 (10)	0.0195 (10)	0.0006 (9)	0.0069 (8)	−0.0020 (9)
C24	0.0215 (11)	0.0189 (10)	0.0240 (11)	−0.0003 (9)	0.0078 (9)	−0.0021 (9)
O25	0.0245 (8)	0.0198 (8)	0.0236 (8)	−0.0008 (6)	0.0045 (6)	0.0030 (6)
C26	0.0262 (12)	0.0242 (11)	0.0318 (12)	−0.0044 (10)	0.0099 (10)	0.0003 (10)
N27	0.0240 (10)	0.0326 (11)	0.0350 (11)	−0.0106 (9)	0.0074 (9)	0.0000 (9)
C28	0.0256 (12)	0.0330 (13)	0.0260 (12)	−0.0037 (10)	0.0044 (10)	−0.0007 (10)
C29	0.0209 (10)	0.0261 (11)	0.0213 (10)	−0.0020 (9)	0.0074 (9)	−0.0039 (9)
C30	0.0243 (11)	0.0282 (12)	0.0196 (10)	−0.0045 (10)	0.0003 (9)	−0.0004 (9)
O31	0.0329 (9)	0.0399 (10)	0.0267 (8)	−0.0085 (8)	−0.0057 (7)	0.0034 (8)
C32	0.0332 (14)	0.0317 (13)	0.0471 (16)	−0.0090 (11)	0.0083 (12)	0.0121 (12)
N33	0.0265 (10)	0.0259 (10)	0.0250 (10)	−0.0084 (8)	0.0035 (8)	0.0008 (8)
O34	0.0397 (10)	0.0207 (8)	0.0301 (9)	0.0015 (7)	0.0116 (8)	0.0012 (7)
O35	0.0469 (11)	0.0349 (10)	0.0232 (8)	0.0128 (8)	0.0175 (8)	0.0032 (7)
N36	0.0275 (10)	0.0190 (9)	0.0227 (10)	0.0016 (8)	0.0101 (8)	0.0030 (7)
O37	0.0326 (9)	0.0322 (9)	0.0234 (8)	0.0104 (7)	0.0094 (7)	0.0086 (7)
O38	0.0265 (9)	0.0322 (9)	0.0323 (9)	0.0096 (7)	0.0111 (7)	0.0091 (7)
O39	0.0335 (10)	0.0616 (14)	0.0390 (11)	0.0035 (9)	0.0009 (8)	−0.0149 (10)
O40	0.0599 (13)	0.0568 (13)	0.0304 (10)	−0.0153 (11)	0.0161 (9)	0.0023 (9)
N41	0.0272 (11)	0.0426 (13)	0.0229 (10)	0.0009 (9)	0.0060 (8)	−0.0030 (9)
O42	0.0497 (12)	0.0437 (12)	0.0462 (12)	0.0081 (10)	0.0199 (10)	0.0034 (9)
O43	0.0491 (13)	0.0671 (15)	0.0602 (14)	0.0178 (11)	0.0264 (11)	−0.0122 (12)

### Geometric parameters (Å, °)

**Table:**

Co1—O2	2.1228 (15)	C19—N33	1.336 (3)
Co1—N6	2.0878 (18)	N20—N21	1.375 (2)
Co1—O16	2.0000 (15)	N20—H9	0.861
Co1—O18	2.1232 (16)	N21—C22	1.290 (3)
Co1—N21	2.0763 (18)	C22—C23	1.458 (3)
Co1—O25	2.0044 (16)	C22—H221	0.931
O2—C3	1.249 (3)	C23—C24	1.423 (3)
C3—N4	1.327 (3)	C23—C29	1.422 (3)
C3—N5	1.369 (3)	C24—O25	1.295 (3)
N4—H3	0.864	C24—C26	1.424 (3)
N4—H6	0.875	C26—N27	1.334 (3)
N5—N6	1.368 (2)	C26—C32	1.485 (3)
N5—H2	0.861	N27—C28	1.348 (3)
N6—C7	1.287 (3)	N27—H271	0.859
C7—C8	1.457 (3)	C28—C29	1.372 (3)
C7—H71	0.937	C28—H324	0.935
C8—C9	1.419 (3)	C29—C30	1.504 (3)
C8—C15	1.425 (3)	C30—O31	1.421 (3)
C9—C10	1.515 (3)	C30—H301	0.970
C9—C12	1.370 (3)	C30—H302	0.964
C10—O11	1.430 (3)	O31—H17	0.814
C10—H101	0.988	C32—H321	0.964
C10—H102	0.990	C32—H322	0.959
O11—H325	0.818	C32—H323	0.961
C12—N13	1.354 (3)	N33—H4	0.856
C12—H121	0.977	N33—H5	0.878
N13—C14	1.334 (3)	O34—H7	0.834
N13—H131	0.753	O34—H19	0.833
C14—C15	1.421 (3)	O35—N36	1.248 (2)
C14—C17	1.491 (3)	N36—O37	1.251 (2)
C15—O16	1.302 (3)	N36—O38	1.253 (2)
C17—H172	0.965	O39—H11	0.844
C17—H173	0.967	O39—H45	0.847
C17—H23	0.955	O40—N41	1.262 (3)
O18—C19	1.245 (3)	N41—O42	1.252 (3)
C19—N20	1.371 (3)	N41—O43	1.219 (3)
			
O2—Co1—N6	76.54 (6)	C14—C17—H23	111.7
O2—Co1—O16	154.44 (7)	H172—C17—H23	109.2
N6—Co1—O16	85.09 (7)	H173—C17—H23	107.6
O2—Co1—O18	83.21 (6)	Co1—O18—C19	113.69 (14)
N6—Co1—O18	110.63 (7)	O18—C19—N20	120.60 (19)
O16—Co1—O18	86.89 (6)	O18—C19—N33	123.3 (2)
O2—Co1—N21	100.41 (6)	N20—C19—N33	116.08 (19)
N6—Co1—N21	171.12 (7)	C19—N20—N21	116.04 (17)
O16—Co1—N21	100.21 (7)	C19—N20—H9	122.1
O18—Co1—N21	76.98 (6)	N21—N20—H9	121.8
O2—Co1—O25	94.13 (6)	Co1—N21—N20	112.65 (13)
N6—Co1—O25	85.04 (7)	Co1—N21—C22	129.72 (15)
O16—Co1—O25	101.92 (7)	N20—N21—C22	117.59 (18)
O18—Co1—O25	162.82 (6)	N21—C22—C23	123.5 (2)
N21—Co1—O25	86.88 (7)	N21—C22—H221	117.8
Co1—O2—C3	113.95 (13)	C23—C22—H221	118.6
O2—C3—N4	123.8 (2)	C22—C23—C24	122.8 (2)
O2—C3—N5	120.31 (19)	C22—C23—C29	117.9 (2)
N4—C3—N5	115.94 (19)	C24—C23—C29	119.3 (2)
C3—N4—H3	119.3	C23—C24—O25	125.6 (2)
C3—N4—H6	120.1	C23—C24—C26	118.2 (2)
H3—N4—H6	120.6	O25—C24—C26	116.2 (2)
C3—N5—N6	116.22 (18)	Co1—O25—C24	129.50 (14)
C3—N5—H2	121.6	C24—C26—N27	118.6 (2)
N6—N5—H2	122.1	C24—C26—C32	121.5 (2)
Co1—N6—N5	112.70 (13)	N27—C26—C32	119.9 (2)
Co1—N6—C7	128.37 (15)	C26—N27—C28	124.9 (2)
N5—N6—C7	117.54 (18)	C26—N27—H271	117.8
N6—C7—C8	122.68 (19)	C28—N27—H271	117.4
N6—C7—H71	119.1	N27—C28—C29	119.8 (2)
C8—C7—H71	118.2	N27—C28—H324	119.4
C7—C8—C9	118.30 (19)	C29—C28—H324	120.8
C7—C8—C15	122.33 (19)	C23—C29—C28	119.2 (2)
C9—C8—C15	119.37 (19)	C23—C29—C30	121.9 (2)
C8—C9—C10	123.38 (19)	C28—C29—C30	118.9 (2)
C8—C9—C12	119.5 (2)	C29—C30—O31	109.48 (19)
C10—C9—C12	117.05 (19)	C29—C30—H301	108.6
C9—C10—O11	114.45 (18)	O31—C30—H301	109.9
C9—C10—H101	108.5	C29—C30—H302	110.4
O11—C10—H101	108.5	O31—C30—H302	108.9
C9—C10—H102	108.7	H301—C30—H302	109.6
O11—C10—H102	106.7	C30—O31—H17	104.6
H101—C10—H102	110.0	C26—C32—H321	110.3
C10—O11—H325	106.4	C26—C32—H322	108.3
C9—C12—N13	119.7 (2)	H321—C32—H322	109.9
C9—C12—H121	121.5	C26—C32—H323	109.5
N13—C12—H121	118.8	H321—C32—H323	110.0
C12—N13—C14	124.19 (19)	H322—C32—H323	108.8
C12—N13—H131	118.4	C19—N33—H4	117.6
C14—N13—H131	117.4	C19—N33—H5	118.7
N13—C14—C15	119.4 (2)	H4—N33—H5	123.5
N13—C14—C17	119.5 (2)	H7—O34—H19	107.0
C15—C14—C17	121.12 (19)	O35—N36—O37	119.20 (19)
C8—C15—C14	117.81 (19)	O35—N36—O38	121.04 (19)
C8—C15—O16	125.55 (19)	O37—N36—O38	119.76 (18)
C14—C15—O16	116.61 (19)	H11—O39—H45	108.0
Co1—O16—C15	127.43 (14)	O40—N41—O42	115.9 (2)
C14—C17—H172	109.0	O40—N41—O43	122.6 (2)
C14—C17—H173	109.9	O42—N41—O43	121.4 (2)
H172—C17—H173	109.3		

### Hydrogen-bond geometry (Å, °)

**Table:**

*D*—H···*A*	*D*—H	H···*A*	*D*···*A*	*D*—H···*A*
C7—H71···O11	0.94	2.33	2.992 (3)	127
C17—H172···O11^i^	0.97	2.58	3.331 (3)	135
C22—H221···O2^ii^	0.93	2.35	3.153 (3)	144
N27—H271···O35^iii^	0.86	2.18	2.950 (3)	149
N27—H271···N36^iii^	0.86	2.57	3.400 (3)	163
N27—H271···O37^iii^	0.86	2.27	2.995 (3)	142
C30—H301···O37^iv^	0.97	2.49	3.369 (3)	151
O11—H325···O16^i^	0.82	2.00	2.783 (3)	159
O11—H325···O18^i^	0.82	2.59	3.132 (3)	125
O39—H11···O40	0.84	2.01	2.851 (3)	175
O31—H17···O42^v^	0.81	1.95	2.718 (3)	158
C17—H23···O40	0.96	2.59	3.391 (3)	141
O39—H45···O31^vi^	0.85	2.05	2.892 (3)	171
O34—H7···O11^vii^	0.83	1.96	2.767 (3)	164
O34—H19···C24	0.83	2.54	3.149 (3)	131
O34—H19···O25	0.83	2.06	2.852 (3)	158
N13—H131···O40	0.75	2.22	2.963 (3)	169
N13—H131···O42	0.75	2.38	2.971 (3)	136
N5—H2···O38^i^	0.86	1.93	2.789 (3)	174
N4—H3···O37^viii^	0.86	2.09	2.948 (3)	174
N33—H4···O39^ix^	0.86	2.08	2.893 (3)	158
N33—H5···O34^ii^	0.88	2.06	2.874 (3)	153
N4—H6···O35^i^	0.88	2.09	2.967 (3)	174
N20—H9···O34^ii^	0.86	2.21	2.946 (3)	143

Symmetry codes: (i) −*x*+2, −*y*+1, −*z*+1; (ii) −*x*+2, −*y*+1, −*z*; (iii) −*x*+1, *y*−1/2, −*z*+1/2; (iv) *x*, −*y*+3/2, *z*−1/2; (v) *x*, *y*, *z*−1; (vi) −*x*+1, −*y*+1, −*z*; (vii) *x*, −*y*+1/2, *z*−1/2; (viii) −*x*+2, *y*−1/2, −*z*+1/2; (ix) *x*+1, *y*, *z*.
